# Does Lumbar Puncture Still Have Clinical Value for Patients with Amyotrophic Lateral Sclerosis?

**DOI:** 10.3390/brainsci15030258

**Published:** 2025-02-27

**Authors:** Federica Ginanneschi, Stefania Casali, Chiara Cioni, Delia Righi, Emanuele Emmanuello, Cecilia Toccaceli, Domenico Plantone, Nicola De Stefano

**Affiliations:** Department of Medical, Surgical and Neurological Sciences, University of Siena, 53100 Siena, Italy; stefania.casali1@gmail.com (S.C.); cioni5@unisi.it (C.C.); d.righi4@student.unisi.it (D.R.); e.emmanuello94@gmail.com (E.E.); c.toccaceli@student.unisi.it (C.T.); domenico.plantone@unisi.it (D.P.); destefano@unisi.it (N.D.S.)

**Keywords:** ALS, biomarkers, blood–brain barrier, cerebrospinal fluid, prognosis

## Abstract

**Background:** The relationship between routine cerebrospinal fluid (CSF) testing and clinical and prognostic data in amyotrophic lateral sclerosis (ALS) remains unclear. Additionally, biochemical data have never been correlated with markers of neurodegeneration. The purpose of this study is to determine whether lumbar puncture may still have clinical utility in ALS. **Methods:** We collected the CSF profiles of 140 ALS subjects. CSF protein, albumin, IgG, IgG index, albumin quotient (QAlb), t-tau, p-tau, and Aβ42 were analyzed. **Results:** Approximately one-quarter of ALS patients had elevated levels of protein, albumin, and QAlb in the CSF, but these were not associated with clinical or survival data. Among the neurodegeneration markers, the percentage of patients with abnormal values ranged from 26.3% to 35.4%. The p-tau/t-tau ratio and Aβ42 were correlated with both the ALS progression rate and the time from diagnosis to death. Aβ42 was the prognostic marker most strongly associated with survival. **Conclusions:** The lack of correlation between biochemical CSF findings and the clinical and/or prognostic status of ALS suggests that these markers have no clinical value. However, neurodegeneration markers that are easily measurable in clinical laboratories, particularly Aβ42, may be useful at the time of diagnosis for predicting ALS survival and progression rate.

## 1. Introduction

Amyotrophic lateral sclerosis (ALS) is a heterogeneous disease characterized by diverse genetic backgrounds, varied clinical presentations, and distinct histopathological and molecular alterations. These factors contribute to variability in the onset phenotype and progression rate. Traditionally, ALS diagnosis has relied primarily on clinical findings, with electrophysiological data serving as a confirmatory tool. Imaging and laboratory techniques are mainly used in clinical settings to rule out other diseases. While cerebrospinal fluid (CSF) findings rarely alter the diagnosis in suspected ALS cases, lumbar puncture is sometimes still performed as a routine procedure in clinical practice. Strong evidence suggests that the blood–brain barrier (BBB) is compromised in the early stages of ALS [1,2,3] and worsens as the disease progresses [4]. Mitochondrial pathology, aberrant astrocytes, and neuroinflammation observed in ALS may help explain the BBB alterations seen in both patients and experimental models [5]. While numerous studies have demonstrated elevated levels of serum-derived proteins in the CSF and an increased albumin CSF/serum quotient (QAlb) in living ALS patients compared to healthy controls [6,7,8,9], the clinical significance of these findings remains to be fully understood, particularly regarding prognosis. Again, there are no data in the literature regarding a possible correlation between CSF proteins and/or QAlb and other CSF biomarkers, such as total tau (t-tau), phosphorylated tau (p-tau), the p-tau/t-tau ratio, and amyloid beta peptide 42 (Abeta-42). Recent papers support the role of these CSF biomarkers of neurodegeneration as diagnostic and prognostic biomarkers in ALS [10,11,12,13]. Indeed, although these biomarkers do not outperform the predictive value of survival and the diagnostic performance of neurofilaments, they can be easily measured by all laboratories and are now considered valuable biomarkers to integrate into the monitoring procedure as a complement of neurofilaments [12]. The purpose of this study was to explore potential connections between the CSF profile and demographic, laboratory, clinical, and survival data in an ALS cohort from Italy, evaluated at Siena University Hospital.

## 2. Materials and Methods

### 2.1. Subjects

We retrospectively analyzed the medical records of patients diagnosed with ALS and admitted to the Motor Neuron Diseases Center at Siena University Hospital over the past 15 years. ALS was diagnosed by neurologists experienced in the field of neuromuscular diseases based on the El Escorial revised criteria for clinically definite or clinically probable ALS [14]. We collected data obtained during the initial diagnostic work-up at the time of ALS diagnosis. We excluded subjects with comorbidities that could influence CSF data, such as inflammatory/autoimmune diseases affecting the peripheral and central nervous systems, as well as other neurodegenerative diseases apart from ALS. All patients had undergone brain and spinal cord magnetic resonance imaging, extensive nerve conduction studies, screening for neurotropic viruses, and testing for immunological/systemic diseases.

Other exclusion criteria included the absence of clinical and/or demographic data in patient records. We collected data on age at symptom onset, the presence of a family history of ALS, the time from onset to first assessment (i.e., the diagnostic delay, in months), and survival, calculated in months from symptom onset to death. The revised ALS Functional Rating Scale (ALS-FRSr, range 0–48, with lower scores indicating greater disability) [15] was used to measure global disability. The ALS progression rate (DeltaFS) was calculated as 48-ALS-FRSr at the time of diagnosis, divided by the duration (in months) from onset to diagnosis [16]. We examined the forced vital capacity (FVC) of subjects. We recorded whether the symptoms began in the spinal or bulbar areas and classified patients into those with bulbar-onset and spinal-onset ALS. Since ALS is unlikely to be diagnosed at the onset of symptoms, we have no choice but to rely on the patient’s medical history to distinguish between patients with bulbar and spinal onset. The patients classified as bulbar onset exhibited dysarthria or dysphagia, fasciculations, tongue wasting, and no peripheral spinal involvement [17]. Those classified as spinal onset presented with cramps, fasciculations, muscle weakness, and atrophy of the limbs and trunk.

### 2.2. Cerebrospinal Fluid Biochemical Analysis

For the analysis, we considered IgG and albumin levels in both CSF and serum, as well as CSF protein. The QAlb was calculated using the formula CSF albumin/serum albumin × 1000. Additionally, we measured the IgG index, which was determined as the quotient of IgG and albumin concentrations in both cerebrospinal fluid and serum. Protein concentration was measured using the Bradford method [18]. The reference normal limits were as follows: 20–50 mg/dL for CSF protein, 10–32 mg/dL for CSF albumin, 0–4 mg/dL for IgG, ≤0.0063 for QAlb, and ≤0.71 for the IgG index. Any biochemical values that exceeded the reference ranges of our laboratory were considered abnormal. We then proceeded to collect the values for t-tau, p-tau, the p-tau/t-tau ratio, and Abeta-42. Establishing the normal range for t-tau is quite challenging, as inconclusive results have been published regarding its age-dependency in healthy individuals [19]. It is known that the discriminative power is high in younger individuals but decreases in older individuals due to neuronal loss during normal aging and the redistribution of soluble t-tau from the brain into the CSF [19]. For these reasons, and based on the normative values from Sjögren’s study involving a large population of 231 healthy individuals [20], we considered it reasonable to define a reference range of 80–450 pg/mL for CSF t-tau. Additionally, separate reference values for different age groups were established: <275 pg/mL for individuals aged ≤70 years old and <450 pg/mL for those over 70 years old. CSF-Abeta42 and p-tau did not correlate with age [21], and according to literature and local laboratory references, the cut-offs were set to >450 pg/mL and ≤55 pg/mL, respectively [21].

### 2.3. Statistical Analysis

The continuous variables (age at onset, delay of diagnosis, DeltaFS, ALS-FRSr, FVC, and elapsed time from diagnosis to death) were assessed for normality using the Kolmogorov-Smirnov test with Lilliefors correction. Descriptive statistics for continuous variables are presented as median and interquartile range (IQR). Differences in continuous variables were analyzed using the Mann–Whitney test. The categorical variables were dichotomized: female/male = 0/1; spinal onset/bulbar onset = 0/1. For categorical variables, we used Fisher’s exact test if at least in one cell the number of observations was equal to or less than 5; otherwise, we used the χ^2^ test. Correlations between CSF data were examined using Spearman’s correlation coefficient.

At this stage, we conducted three logistic regression analyses, with “normal/abnormal” t-tau, p-tau, and Abeta-42 as the dependent variables, and age at ALS onset, gender, diagnosis delay, bulbar/spinal onset, DeltaFS, survival, and ALS-FRSr as the independent variables. The multivariate logistic regression analysis was performed using variables with a liberal significance level of *p* ≤ 0.2 [22,23]. The eligible variables for the multivariable logistic regression were tested using Spearman’s rho (rs) to prevent spurious relationships. If the correlation coefficient (rs) was ≥0.70, only one variable was selected for further use in the multivariable analysis based on clinical significance. A stepwise, backward LR multivariable logistic regression analysis was then performed with the remaining determinants.

For survival analyses, Kaplan–Meier curves were generated for normal/abnormal CSF values. Differences between the curves were assessed using the log-rank test. Right-censored data were included in the analysis. All tests were performed with GraphPad Prism 7. Statistical significance was considered at *p*-value < 0.05.

## 3. Results

We included 140 ALS patients in the study, consisting of 83 (59%) males and 57 (40.7%) females, with a median age of 67 years old (range 24–88 years old), from 140 unrelated families. The demographic and clinical features are detailed in Table 1.

Table 2 shows the CSF parameters. Approximately a quarter of ALS patients exhibit elevated levels of CSF protein and albumin. The QAlb was elevated in 29.9% of subjects. An increase in QAlb was more common in males than in females (40% vs. 15%, respectively). Males also had higher levels of CSF proteins compared to females (*p* = 0.006). Only 2.1% of subjects had an abnormal IgG index. Among the neurodegeneration markers, the percentage of patients with altered values ranges from 26.3% to 35.4%, with t-tau being the most frequently altered. Specifically, we observed an increase in t-tau and p-tau, and a decrease in Abeta42. No significant differences were found between males and females regarding t-tau, p-tau, and Abeta42.

When comparing patients with bulbar onset to those with spinal onset, as expected, we observed a significantly higher progression rate (DeltaFS) of ALS in patients with bulbar onset. However, no significant differences in CSF parameters were observed between the two groups (Table 3).

Table 4 presents the correlation analyses. CSF protein, IgG, and QAlb do not correlate with either clinical data or neurodegeneration biomarkers. However, the analysis of neurodegeneration markers in relation to clinical parameters in ALS patients reveals several notable correlations. Firstly, the age of disease onset correlates with t-tau and p-tau levels. Survival, calculated as the number of months elapsing from symptom onset to death, correlates with several markers of neurodegeneration, specifically, t-tau Abeta42, and the p-tau/t-tau ratio. The progression rate of ALS, as measured using the ALS-FRSr scale, correlates with Abeta-42 levels. The clinical severity of ALS, as assessed using the DeltaFS score, correlates only with Abeta-42 levels and p-tau/t-tau ratio.

The results of the univariate analysis for each independent variable and the simultaneous multivariable regression analysis are presented in Table 5 for t-tau and in Table 6 for Abeta42. Taking into account p-tau, in the univariate analysis, a *p*-value ≤ 0.2 was reached only for the “age at onset” (*p* = 0.0016), so the multivariable regression analysis was not performed. The significant determinants (*p* < 0.05) in the simultaneous multivariable regression analysis were “survival” and “ALS-FRSr” for Abeta-42 (Table 6B). Regarding t-tau, no independent variable was found to be significant in multivariable regression analysis (Table 5B).

**Figure 1:**
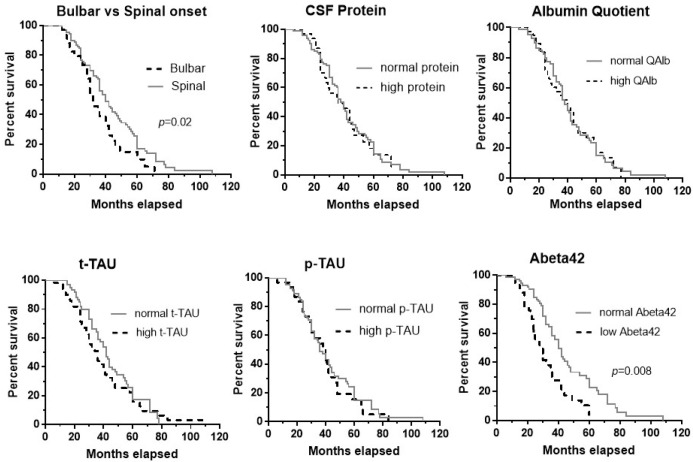
Kaplan–Meier curves for bulbar vs. spinal onset, and normal/abnormal values of the following CSF measurements: protein, albumin quotient, t-tau, p-tau, and Abeta42. There was a significant effect on survival of bulbar ALS onset (*p* = 0.02) and abnormal Abeta42 values (*p* = 0.0008).

Figure 1 shows the median survival time for bulbar vs. spinal onset (32 vs. 40 months), impaired/normal CSF protein (36 vs. 38 months), QAlb (38 vs. 40 months), t-tau (36 vs. 42 months), p-tau (36 vs. 40 months), and Abeta42 (30 vs. 40 months). There was a significant effect on survival for bulbar ALS onset (Log-rank test, Chi-square = 5.25, *p* = 0.02) and abnormal Abeta42 values (Log-rank test, Chi-square = 11.22, *p* = 0.0008). The survival time for males vs. females was similar.

## 4. Discussion

Before presenting our results, we would like to underline that our study has several limitations. First, it is retrospective and involves only a single cohort, which may limit the generalizability of the results. Second, it lacks a control group of patients with other neurodegenerative diseases. Finally, we do not have longitudinal data on CSF biomarkers.

Blood–brain barrier damage can occur in many neurodegenerative diseases, including ALS [24]. Post-mortem, clinical, and experimental studies provide strong evidence of BBB hyperpermeability prior to or at the very early stages of ALS symptom onset [25,26], and it is likely that the mechanisms underlying BBB dysfunction are also involved in ALS etiopathogenesis [3,5]. In fact, in the experimental ALS model, restoration of BBB integrity delayed motor neuron dysfunction and death [26,27].

Measurements of CSF protein, CSF albumin, and the CSF/serum quotient (QAlb) have confirmed the dysfunction of the BBB in living ALS patients [7,26,28]. Several studies have been conducted to determine if there is a correlation between the extent of BBB damage and the prognosis of ALS, with controversial results [7,8,9,28,29]. In particular, the increase in CSF proteins and other BBB injury indices is reported to be related to reduced survival in some studies, but this is not confirmed by others. To add to the complexity, the relationship between BBB damage and poor ALS prognosis is sometimes described as being dependent on sex and ALS phenotype [8,9,28]. The inconsistent literature findings regarding the prognostic value of CSF protein may depend on inherent differences among the cohorts studied.

In our cohort, similarly to another previously described Italian cohort [30], BBB damage can be seen in approximately 25% of patients, but it does not correlate with clinical or survival data. As expected, patients with the bulbar phenotype have lower survival than those with the spinal phenotype; however, the protein content of the fluid does not differ between the two groups.

Our results do not, of course, contradict the role that BBB damage plays in the pathogenesis of ALS. The lack of correlation between BBB impairment and clinical data could be explained by the fact that BBB damage occurs early in the disease course, during the pre-symptomatic stages of ALS [1,2,3]. Therefore, it may lose prognostic significance as the disease progresses. In addition, it is noteworthy that there are other biomarkers associated with BBB damage in ALS, such as matrix metalloproteinases and S100B, that could also serve as prognostic indicators.

The fact that BBB dysfunction is triggered by mechanisms closely linked to ALS aetiopathogenesis has generated interest in its diagnostic and therapeutic potential [3,5]. Strategies focusing on repairing and protecting the BBB, improving drug delivery, and modulating neurovascular interactions hold promise for ALS patients.

In post-mortem studies, changes related to BBB breakdown are identified in both familial and sporadic ALS [31]. However, while BBB damage has been observed in some familiar ALS models (e.g., SOD1 mutations, [4]), the extent and mechanisms of BBB dysfunction may differ compared to sporadic ALS.

Unlike previous studies, we decided to explore the correlations between CSF proteins and t-tau, p-tau, and Abeta42. These are unanimously viewed as well-established biomarkers for Alzheimer’s disease, and their use as diagnostic and prognostic biomarkers in ALS is under discussion [32,33]. Interestingly, a recent metanalysis supports the role of t-tau and p-tau as ALS diagnostic biomarkers [11].

Our findings indicate that t-tau, p-tau, Abeta42, the CSF protein profile, and the QAlb ratio are not linked in any way, supporting the idea that the CSF protein profile cannot be considered in establishing either the prognosis or the progression rate of ALS.

On the other hand, these biomarkers, principally the reduction of p-tau/t-tau ratio and Abeta42, were correlated with both ALS progression rate and the time elapsed from diagnosis to death. Moreover, a relationship between Abeta42 and the ALS-FRSr score has been shown. The Kaplan–Meier estimator of the probability of death over time showed that Abeta42 is the prognostic index most closely related to survival in ALS subjects, at least in our cohort. Many previous studies suggest that increased t-tau and decreased p-tau/t-tau ratio may be used as predictive or diagnostic ALS biomarkers [11,32,34,35,36,37], whereas the clinical significance of Abeta42 is more debated [38]. The amyloid precursor protein and its proteolytic fragments have emerged not only as drivers of Alzheimer’s disease but also as one of the earliest signatures in ALS, preceding or anticipating neuromuscular junction instability and muscle denervation [38]. It is known that Abeta peptide accelerates the onset of motor impairment in ALS neurons [39]; accordingly, increased Abeta peptide immunoreactivity has been reported in the anterior horn neurons of subjects with familial and sporadic ALS [40,41]. Among living ALS patients, reduced CSF Abeta42 concentrations have been associated with shorter survival times [34,42], although one study suggests that its utility is limited solely to diagnostic purposes [37]. Notably, Steinacker et al. [43] demonstrated an inverse correlation between beta-amyloid precursor protein and neurofilaments in ALS patients with rapid disease progression, suggesting that low CSF concentrations of this protein are associated with extensive neuro-axonal damage.

While Abeta42 is not generally regarded as a key driver of ALS, its involvement in inflammation, neurotoxicity, and protein aggregation suggests that it may contribute to both disease progression and reduced survival. Specifically, evidence suggests that Abeta42 may play a role in the cellular response to axonal damage, and that its intracellular deposition could lead to oxidative stress, activation of pro-apoptotic pathways, and TDP-43 accumulation [42,43]. In our cohort, nearly one-third of the patients exhibited low Abeta42 levels, and these levels correlated with both survival and clinical status, supporting a dual diagnostic and prognostic role for Abeta42 in ALS.

## 5. Conclusions

Our study, conducted on a relatively large ALS population, indicates that CSF biochemical findings related to BBB dysfunction do not correlate with clinical status or prognosis in ALS patients. Therefore, these findings seem to have limited clinical value. However, neurodegeneration markers that are easily measurable by most clinical laboratories, particularly Abeta42 and the p-tau/t-tau ratio, show promise as prognostic tools. These markers may be useful at the time of diagnosis to predict survival and disease progression rates in ALS patients.

Further research is warranted to validate these findings and explore the potential of these markers for improving clinical management, and personalized medicine approaches for ALS patients.

## Figures and Tables

**Table 1:** Demographic and clinical features of the ALS cases.

Demographic/Clinical Features at Diagnosis	Cases (n. 140)
Gender M vs. F (n., %)	83 [59.3%] vs. 57 (40.7%)
Age onset, median [10th–90th]	67 [48.7–77.3]
Diagnosis delay in months, median [10th–90th]	11 [6–21.5]
DeltaFS, median [10th–90th]	0.58 [0.2–1.88]
Bulbar onset vs. Spinal onset (n., %)	40 (28.6%) vs. 100 (71.4%)
ALS-FRSr score, median [10th–90th]	41 [31.3–45]
FVC %, median [10th–90th]	85 [43.3–110]
Survival in months, median [10th–90th]	36 [17–64.5]

ALS: amyotrophic lateral sclerosis; ALS-FRSr: ALS Functional Rate Scale, revised; Delta FS: (48 − ALS-FRSr score)/time from symptom onset to evaluation (months); F: female; FVC: forced vital capacity; M: male; n.: number; 10th–90th refers to the percentile.

**Table 2:** Cerebrospinal fluid parameters.

Biochemical Parameters	Cases (n. 140)
Protein (mg/dL), median [10th–90th]	40 [25–72.2]
Normal	74.6%
Elevated	25.4%
above 70 mg/dL	10%
above 100 mg/dL	4.3%
Albumin (mg/dL), median [10th–90th]	24 [15–51.2]
Normal	73.7%
Elevated	26.3%
QAlb, median [10th–90th]	0.0066 [0.0043–0.014]
Normal	70.1%
Elevated	29.9%
IgG (mg/dL), median [10th–90th]	2.6 [1.6–6]
Normal	79.6%
Elevated	20.4%
t-tau (pg/mL), median [10th–90th]	294 [126–573]
Normal	64.6%
Elevated	35.4%
p-tau (pg/mL), median [10th–90th]	40 [22.3–72]
Normal	73.7%
Elevated	26.3%
Abeta-42 (pg/mL), median [10th–90th]	635 [308–1078]
Normal	70.7%
Low	29.3%

QAlb: albumin quotient, calculated with the formula: CSF albumin/serum albumin × 1000. 10th–90th refers to the percentiles. n.: number.

**Table 3:** Cerebrospinal fluid parameters in bulbar vs. spinal onset.

	Bulbar Onset (n. 40)	Spinal Onset (n. 100)	*p*-Value
Age onset, median [10th–90th]	68 [45.7–81]	66.5 [50.8–77]	n.s.
Gender (M/F, n.)	16/22	65/33	0.0098
DeltaFS, median [10th–90th]	0.75 [0.33–2.1]	0.54 [0.17–1.8]	0.037
ALS-FRSr score, median [10th–90th]	40.5 [22.9–45]	41 [32–45.5]	n.s.
Protein (mg/dL), median [10th–90th]	37.7 [23.7–87.2]	40 [28.1–72.3]	n.s.
Albumin (mg/dL), median [10th–90th]	21.8 [13.5–41]	24.8 [15.8–56.7]	n.s.
IgG (mg/dL), median [10th–90th]	2.6 [1.6–10.9]	2.6 [1.7–5.3]	n.s.
t-TAU (pg/mL), median [10th–90th]	324.5 [102–680]	277.5 [126–510]	n.s.
p-TAU (pg/mL), median [10th–90th]	39.5 [16.7–75.7]	40.5 [21.9–68]	n.s.
Abeta-42 (pg/mL), median [10th–90th]	583 [271–1069]	621 [314–1064]	n.s.

ALS-FRSr: ALS Functional Rate Scale, revised; Delta FS: (48 − ALS-FRSr score)/time from symptom onset to evaluation (months); F: female; n.: number; n.s.: not significant; 10th–90th refers to the percentiles.

**Table 4:** Correlation analyses among the CSF parameters.

Spearman’s Rho	Age Onset	DeltaFS	ALS-FRSr	Survival	t-tau	p-tau	Abeta-42	p-tau/t-tau
CSF protein	n.s.	n.s.	n.s.	n.s.	n.s.	n.s.	n.s.	n.s.
CSF IgG	n.s.	n.s.	n.s.	n.s.	n.s.	n.s.	n.s.	n.s.
Qalb	n.s.	n.s.	n.s.	n.s.	n.s.	n.s.	n.s.	n.s.
t-tau	r = 0.34 *p* = 0.0003	n.s.	n.s.	r = −0.23 *p* = 0.016		r = 0.75 *p* < 0.0001	n.s.	r = −0.51 *p* < 0.0001
p-tau	r = 0.338 *p* = 0.0004	n.s.	n.s.	n.s.	r = 0.75 *p* < 0.0001		r = −0.314 *p* = 0.009	n.s.
Abeta-42	n.s.	r = −0.23 *p* = 0.016	r = 0.23 *p* = 0.016	r = 0.22 *p* = 0.02	n.s.	r = 0.314 *p* = 0.0009		n.s.
p-tau/t-tau	n.s.	r = 0.22 *p* = 0.02	n.s.	r = 0.26 *p* = 0.006	r = −0.51 *p* < 0.001	r = 0.31 *p* = 0.0009	n.s.	

ALS-FRSr: ALS Functional Rate Scale, revised. CSF: cerebrospinal fluid; Delta FS: (48 − ALS-FRSr score)/time from symptom onset to evaluation (months); n.s.: not significant; QAlb: albumin quotient, calculated with the formula: CSF albumin/serum albumin × 1000. Survival is calculated in months.

**Table 5:** **A.** t-tau. Demographic and clinical differences between the group of patients with impaired test results and those with normal test results. **B.** t-tau. Multivariable logistic regression to determine which variables play a crucial role in predicting the alteration of t-tau.

**A**					
**t-tau**		**Impaired (35.4%)**		**Normal (64.6%)**	***p*-Value**
Age onset, median [10th–90th]		68 [55.7–81]		66.5 [43.9–76]	0.02
Gender (M/F, %)		53.7/46.3		56.6/43.4	0.8
DeltaFS, median [10th–90th]		0.61 [0.2–2.2]		0.56 [0.18–1.99]	0.65
Survival, median [10th–90th]		28 [12.6–62]		36 [17.6–64.8]	0.04
Diagnosis delay (Mth), median [10th–90th]		9 [6–19.1]		12 [6–22.5]	0.027
Bulbar/Spinal (%)		33.3/66.7		26/74	0.5
ALS-FRSr score, median [10th–90th]		41 [30.2–45]		39.5 [32.9–46]	0.84
**B**					
**Variables**	**β values**	**SE**	**OR**	**95% CI**	***p*-Value**
Age onset	0.021	0.020	0.304	0.981–1.062	0.304
Survival	0.002	0.010	1.002	0.982–1.022	0.86
Diagnosis delay	−0.045	0.035	0.956	0.892–1.025	0.95

ALS-FRSr: ALS Functional Rating Scale-Revised; CI: confidence interval; Delta FS: (48 − ALS-FRSr score)/time from symptom onset to evaluation; F: female; M: male; Mth: months; OR: odds ratio; SE: standard error.

**Table 6:** **A.** Abeta-42. Demographic and clinical differences between the group of patients with impaired test results and those with normal test results. **B.** Abeta-42. Multivariable logistic regression to determine which variables play a crucial role in predicting the alteration of Abeta-42.

**A**					
**Abeta-42**		**Impaired (29.3%)**		**Normal (70.7%)**	***p*-Value**
Age onset, median [10th–90th]		71.5 [47–77]		67 [56–81.5]	0.0475
Gender (M/F, %)		41.8/58.82		63.1/36.9	0.04
DeltaFS, median [10th–90th]		0.97 [0.29–2.67]		0.42 [0.2–1.68]	0.0013
Survival, median [10th–90th]		25 [14.4–52.6]		39 [17.5–72]	0.0020
Diagnosis delay (Mth), median [10th–90th]		10 [6.5–20]		10 [6–24]	0.94
Bulbar/Spinal (%)		32.3/67.7		26.6/73.4	0.62
ALS-FRSr score, median [10th–90th]		41 [23–45]		37 [33.7–46]	0.001
**B**					
**Variables**	**β Values**	**SE**	**OR**	**95% CI**	***p*-Value**
Age onset	0.044	0.026	1.045	0.992–1.100	0.098
Gender	−0.996	0.498	0.369	0.139–0.980	0.053
DeltaFS	−0.553	0.481	0.575	0.224–1.477	0.251
Survival	−0.042	0.021	0.959	0.921–0.998	**0.04**
ALS-FRSr score	−0.127	0.059	0.881	0.784–0.990	**0.033**

ALS-FRSr: ALS Functional Rating Scale-Revised; CI: confidence interval; Delta FS: (48 − ALS-FRSr score)/time from symptom onset to evaluation; F: female; M: male; Mth: months; OR: odds ratio; SE: standard error. Significant values are in bold font.

## Data Availability

The data presented in this study are available on request from the corresponding author due to the fact that the statistical analyses and the data obtained were extrapolated from medical records and reports.
